# A nested randomised trial of the effect of tranexamic acid on intracranial haemorrhage and infarction in traumatic brain injury (CRASH-3 trial intracranial bleeding mechanistic study): Statistical analysis plan

**DOI:** 10.12688/wellcomeopenres.14731.3

**Published:** 2019-11-27

**Authors:** Abda Mahmood, Ian Roberts, Haleema Shakur-Still

**Affiliations:** 1 Clinical Trials Unit, Department of Population Health, London School of Hygiene & Tropical Medicine, London, WC1E7HT, UK

**Keywords:** Traumatic brain injury, intracranial haemorrhage, tranexamic acid, statistical analysis plan

## Abstract

**Background:** The CRASH-3 trial is a randomised trial on the effect of tranexamic acid (TXA) versus placebo on death and disability in traumatic brain injury (TBI). The CRASH-3 intracranial bleeding mechanistic study (IBMS) is a randomised trial nested within the CRASH-3 trial to examine the effect of TXA versus placebo on intracranial bleeding and infarction.

**Methods:** Patients eligible for the CRASH-3 trial, with a GCS of 12 or less or intracranial bleeding on a pre-randomisation CT scan are eligible for the IBMS. The occurrence of intracranial bleeding, infarction, haemorrhagic oedematous lesions, mass effect and haemorrhage evacuation is examined within 28 days of randomisation using routinely collected brain scans. The primary outcome is the volume of intra-parenchymal bleeding in patients randomised within three hours of injury (adjusted for prognostic covariates). Secondary outcomes include a composite “poor” outcome, progressive and new intracranial bleeding, intracranial bleeding after neurosurgery and cerebral infarcts seen up to 28 days post-randomisation. All outcomes will be compared between treatment groups.

**Statistical analyses:** The primary outcome will be analysed using a covariate adjusted linear mixed model. The same analysis will be done separately for patients who undergo haemorrhage evacuation post-randomisation. We will express the effect of TXA on the composite outcome, new and progressive bleeding using relative risks and 95% CIs, and on cerebral infarcts using hazard ratios and 95% CIs. We will conduct sensitivity analyses assuming missing data are MCAR or MNAR.

**Conclusion**: The IBMS will provide information on the mechanism of action of TXA in TBI. This pre-specified statistical analysis plan is a technical extension of the published protocol.

**Trial registration: **The CRASH-3 trial was prospectively registered at the International Standard Randomised Controlled Trials registry (19 July 2011) and ClinicalTrials.gov (25 July 2011). The registries were updated with details for the IBMS on 20 December 2016.

## Introduction

Worldwide over 50 million people experience traumatic brain injury (TBI) every year ^1^. TBI is the leading cause of death and disability in young adults ^2^, particularly in low-income and middle-income countries where rates of road traffic crashes are increasing ^3^. Falls are the most frequent cause of TBI in high-income countries ^4^. Intracranial bleeding is common after TBI, mostly in the first few hours after injury ^5^. The larger the bleed the greater the risk of death and long-term disability ^6^. To improve outcome from this life threatening and potentially disabling condition, effective treatments are needed to reduce intracranial haemorrhage expansion.

The permeability of the blood-brain barrier is compromised after TBI ^7^. Tranexamic acid could penetrate the blood-brain-barrier to enter the cerebrospinal fluid ^8, 9^, inhibit the enzymatic breakdown of fibrin blood clots and reduce intracranial haemorrhage expansion. A recent systematic review identified two completed randomised trials of tranexamic acid in TBI ^10^. When the two trials were combined in a meta-analysis (n=478), there appears to be a statistically significant reduction in intracranial haemorrhage growth (RR 0.75, 95% CI 0.58–0.98; P = 0.03) and mortality (RR 0.63, 95% CI 0.40–0.99; P = 0.05) with tranexamic acid. Neither trial found evidence for an increased risk of infarction with tranexamic acid (RR 0.51, 95% CI 0.20–1.32; P = 0.17) ^11^ (0 infarcts – tranexamic acid group, 3 infarcts – placebo group) ^12^. However, the confidence intervals are wide and the quality of this evidence is low. Therefore, the effect of tranexamic acid on mortality, intracranial bleeding and infarction in TBI remains uncertain.

The CRASH-3 trial, with a planned sample size of 13,000 patients, will be the largest randomised trial into the effect of tranexamic acid in isolated TBI ^13^. The CRASH-3 trial is a prospective, international, multi-centre, parallel group, placebo-controlled randomised trial that examines the effects of tranexamic acid on death and disability in TBI. Patients who are within 8 hours of their TBI and have intracranial bleeding on a computed tomography (CT) scan or a Glasgow Coma Score (GCS) of 12 or less, and no significant extra-cranial bleeding, are potentially eligible for inclusion in the CRASH-3 trial. The original 8 hour time window for recruitment was restricted to 3 hours of injury in 2016 in order to reliably examine the effect of tranexamic acid given soon after injury. Eligible patients are randomly allocated (1:1) to receive tranexamic acid or matching placebo (0.9% sodium chloride). The 1 gram loading dose of the trial treatment is administered by intravenous injection within minutes of randomisation in hospital. The 1 gram maintenance dose is administered by intravenous infusion as soon as the loading dose has completed. Tranexamic acid or placebo are given as an additional treatment to the routine management of TBI. The aims and methods for the CRASH-3 trial are presented in detail elsewhere ^13^.

The CRASH-3 trial is based on the premise that intracranial bleeding contributes to head injury death and disability in patients with TBI. By inhibiting fibrinolysis, tranexamic acid is expected to reduce the extent of intracranial bleeding ^14^. Therefore, we expect to see less intracranial bleeding in head CT scans of patients treated with tranexamic acid, particularly in those treated soon after injury when the risk of haemorrhage expansion is greatest ^5^. On the other hand, tranexamic acid might increase the risk of cerebral thrombosis and infarction in TBI patients, potentially worsening neurological outcome ^15– 17^. In this case, we expect to see more infarcts in patients treated with tranexamic acid, particularly in those treated after a prolonged period after injury when there is an increased risk of thrombotic disseminated intravascular coagulation ^15^.

The CRASH-3 Intracranial Bleeding Mechanistic Study (IBMS) is a randomised trial nested within the CRASH-3 trial and examines the effect of tranexamic acid on intracranial bleeding and infarction (protocol version 1.3 currently in use) ^18^. The IBMS evaluates the effect of tranexamic acid on bleeding expansion using a validated method (ABC/2) ^19^ to measure the total bleeding volume on routinely collected CT scans done soon after randomisation. The blinded data from ≈1,000 patients in the IBMS so far suggests that this scan is done within a mean of 44 hours after randomisation. Bleeding is well visualised on CT in the early stage of injury ^20^. Because infarction takes longer to manifest on CT imaging ^21^, the effect of tranexamic acid on infarction is examined using all routinely collected brain imaging (including magnetic resonance imaging) done within 28 days of randomisation. The IBMS will provide information on the mechanism of action of tranexamic acid in TBI and could facilitate the generalisation of trial results ^22^. This pre-specified statistical analysis plan is a technical extension of the published protocol ^19^.

## Trial methods

The aims and methods for the IBMS are presented in detail elsewhere ^18^.

### Aim

The IBMS aims to examine the mechanism by which tranexamic acid exerts its effects in patients with isolated TBI. Specifically, we will assess the effect of tranexamic acid on intracranial bleeding and infarction.

### Trial design and eligibility criteria

The IBMS is a randomised, placebo-controlled, parallel group, international, multi-centre, double-blind trial nested within the CRASH-3 trial. Patients who fulfil the eligibility criteria for the CRASH-3 trial, with a GCS of 12 or less or intracranial bleeding on a CT scan done before randomisation, are eligible for inclusion in the IBMS.

### Trial registration

The CRASH-3 trial was prospectively registered at the International Standard Randomised Controlled Trials registry ( ISRCTN15088122) on 19 July 2011, and ClinicalTrials.gov on 25 July 2011 ( NCT01402882). The registries were updated with details for the IBMS on 20 December 2016.

### Ethical approval

The UK Medical Research and Ethics Committee and Health Research Authority reviewed the protocol and supporting documents for the IBMS and provided a favourable ethical opinion on 8 June 2016 (Research Ethics Committee Reference 12/EE/0274). All participating UK hospitals have provided Research and Development approvals and letters of access for the IBMS to be conducted at their respective sites. The Malaysian Medical Research and Ethics Committee reviewed the protocol and supporting documents for the IBMS and provided favourable ethical opinion on 16 May 2017 (Reference (25) KKM/NIHSEC/P12-476). All relevant national and local ethical approvals will be gained from additional sites. Favourable ethical opinion was received from the Observational/Interventions Research Ethics Committee at LSHTM on 24 May 2016 (Reference 11535). The relevant Medical Research and Ethics Committees will review important protocol modifications for approval before implementation, and registries updated as appropriate.

### Consent to participate

TBI patients are physically and mentally incapable of providing informed consent to participate in a clinical trial. As acknowledged in the Declaration of Helsinki, patients who are incapable of giving consent are an exception to the general rule of informed consent in clinical trials ^23^. In the CRASH-3 trial, patients are unable to provide consent and so consent is sought from the patient’s relative, legal representative or the responsible clinician. If and when the patient regains capacity to provide informed consent, they are informed about the trial and written consent sought to continue their participation in the trial. If a patient or patient representative declines consent, they are withdrawn from the trial. For patients who were included in the trial but did not regain capacity, written informed consent is sought from a relative or legal representative. Written informed consent from patients, their relatives, legal representatives or the responsible clinician includes consent for the publication of anonymised patient data. The requirements of relevant local and national ethics committees are adhered to at all times.

The CRASH-3 trial includes consent to extract data from patient medical records. Collecting brain imaging data for the IBMS is consistent with the consent procedure used in the CRASH-3 trial. It would be impractical to re-consent patients or relatives/legal representatives to brain imaging, particularly for patients who have deceased or are disabled as a result of their injuries where re-consent would be distressing and unwelcome. The LSHTM and national Ethics Committees extended their approvals to extract brain imaging data from CRASH-3 trial patients without further patient consent. Patients who withdrew from the main CRASH-3 trial would not be included in the IBMS.

### Participating hospitals

The hospitals participating in the IBMS were selected based on the number of patients enrolled in the CRASH-3 trial, the availability of electronic imaging at site and the willingness of the trial principal investigator at site to take part. We invited ten of the highest recruiting CRASH-3 trial hospitals in the United Kingdom (UK) to take part (Queen Elizabeth Hospital, Birmingham; Royal London Hospital; University Hospital Coventry; Salford Royal Hospital; St George’s Hospital, London; King’s College Hospital, London; St Mary’s Hospital, London; Addenbrooke’s Hospital, Cambridge; John Radcliffe Hospital, Oxford, Southmead Hospital, North Bristol). We also invited four hospitals in Malaysia to take part: Hospital Sungai Buloh, Penang General Hospital, Hospital Sultanah Nur Zahirah and Hospital Sultanah Bahiyah. We will report all participating sites in the final results publication.

### Sample size

We originally planned for the IBMS to be conducted in 1,000 CRASH-3 trial patients. This sample size was based on the reduction in bleeding volume seen with tranexamic acid in the CRASH-2 Intracranial Bleeding Sub-study (Perel *et al.,* 2012). We expected a 15% reduction in intracranial bleeding with tranexamic acid (24ml tranexamic acid, 28ml placebo), a correlation of 0.6 between pre- and post-randomisation bleeding volumes, and a standard deviation of 28ml. This gave an unadjusted sample size estimate of 1542, which was reduced to 987 with adjustment (1542*(1-(0.6 ^2^)) (Borm, 2007). 

Due to the large amount of missing data (only around half of patients are scanned both pre-randomisation and post-randomisation), we increased the sample size for the CRASH-3 IBMS to include around 1700 patients. This was the approximate maximum number of patients the scan assessor could feasibly collect data from before the CRASH-3 trial completed recruitment. Using the same expected treatment effect, standard deviation, correlation and baseline adjustment values as the original sample size calculation, this increased power to 95%. (at alpha=0.05) However, this calculation is not adjusted for missing data. If we assume that 47% of patients will be dropped from the analyses, this leaves a study with 901 patients scanned both pre-randomisation and post-randomisation. Using the same standard deviation (adjusted for baseline), correlation and baseline adjustment values as the original sample size calculation, a study with 901 patients would have 76% power to detect the expected treatment effect.

### Interim analyses and unblinding

The treatment allocation is double-blinded such that trial team members, outcome assessors and patients are unaware of whether a trial patient will receive tranexamic acid or placebo. There are no interim analyses planned. The final analysis of the unblinded results will take place after recruitment is complete, the data have been cleaned and the trial database has been locked as per the procedures detailed in the Data Management Plan (DMP) (version 1.0) and protocol ^18^.

### Data management and integrity

All trial data are managed in accord with the IBMS DMP which is stored in the Trial Master File. The DMP working procedures are produced in conjunction with the London School of Hygiene and Tropical Medicine (LSHTM) policies and procedures, the Clinical Trials Unit and trial specific working procedures, and regulatory requirements. The web database was built to comply with ICH-GCP guidelines and uses MySQL for data storage. Hypertext Preprocessor (PHP) was used to develop the dynamic web pages for the user interface.

Data are collected at each participating site and directly uploaded into the web database. A number of computerised validation checks have been built into the database to ensure all required fields are complete and irregular entries are flagged. In rare cases of poor internet connection or inadequate facilities, paper versions of the Case Report Forms (CRFs) are completed and transcribed into the web database as soon as possible. A delegate cross-checks the transcription between paper and web CRFs and any detected errors are amended on paper and/or web CRFs immediately. Any revisions to a submitted form are saved automatically in a database log with details of who edited the data and when edits were made. Any changes made from the initial form submission are highlighted in each amended version of a form. All other data checks and cleaning are performed by the IBMS lead. This includes using a download report facility within the database to review the data for inconsistencies and resolve queries as per the procedures detailed in the DMP. The final database lock will take place at the end of the trial within three months of the end of data collection. Data will be exported for statistical analysis in Stata Version 15 [StataCorp LP, College Station, Texas, USA].

### Primary outcome

The mean volume of intra-parenchymal bleeding will be compared between trial arms in patients randomised within three hours of injury, adjusting for prognostic covariates.

In the original IMBS protocol ^18^, we said the total volume of intracranial bleeding would be compared between treatment groups. Since publishing the protocol, we have collected blinded data from 1700 trial patients, which suggest that any effect of tranexamic acid on intracranial bleeding expansion may only be reliably detected in intra-parenchymal bleeds. Intra-parenchymal bleeds are less likely to be surgically evacuated compared to subdural and epidural bleeds, which are often larger and therefore substantially increase intracranial pressure and require urgent neurosurgical evacuation. Large subdural and epidural bleeds are easier to evacuate because they occur outside of the brain tissue, whereas intra-parenchymal bleeds often occur deep within the brain tissue so it is difficult to evacuate them without causing further harm. Therefore, we may not be able to reliably examine the effect of tranexamic acid on subdural and epidural bleed expansion given that large bleeds are often evacuated before we can examine any effect of tranexamic acid on them. Including bleeds that may not be affected by tranexamic acid in the primary outcome would dilute any effect of tranexamic acid on intracranial bleeding expansion to the null. Furthermore, when excluding patients who have undergone neurosurgery by the first rated post-randomisation scan, the proportional expansion of intra-parenchymal bleeding from pre- to post-randomisation is greater than for all other types of intracranial bleeding. Indeed, a recent randomised trial found a statistically significant reduction in intracerebral bleeding expansion with tranexamic acid ^24^. Finally, intra-parenchymal bleeds are often spherical in shape, so there is less measurement error with the ABC/2 method of volume estimation compared to subdural and epidural bleeds, which have concave and convex shapes, respectively. For these reasons, the primary outcome will examine the effect of tranexamic acid on the total volume of intra-parenchymal bleeding.

In the original IBMS protocol ^18^, the primary outcome included all patients randomised within 8 hours of injury. Since the protocol was published, an individual patient data meta-analysis was published which included 40,138 patients with acute severe bleeding enrolled in randomised trials of tranexamic acid ^25^. This meta-analysis showed that immediate treatment improved the odds of survival by more than 70% (OR 1·72, 95% CI 1·42–2·10; p<0·0001). Thereafter, the survival benefit decreased by about 10% for every 15 minutes of treatment delay until 3 hours, after which there was no benefit. To quantify any reduction in bleeding volume with tranexamic acid compared to placebo in the IBMS, we must examine the primary outcome during the interval where bleeding is at greatest risk of expansion. If there is a minimal change in bleeding volume after three hours of injury, including patients treated after three hours of injury in the primary analysis will dilute any effect of tranexamic acid towards the null. Therefore, we will restrict the analysis of the primary outcome to three hours of injury.

### Secondary outcomes

(a) Frequency and volume of progressive bleeding in patients randomised within 3 hours of injury: number of patients with a post-randomisation scan with a total bleeding volume of more than 25% of the volume on the pre-randomisation scan;
(b) Frequency and volume of new bleeding in patients randomised within 3 hours of injury: number of patients with haemorrhage on the post-randomisation scan that was not seen on the pre-randomisation scan;
(c) Number of patients with cerebral infarcts seen on a post-randomisation scan and not known to be present pre-randomisation;
(d) Mean volume of intracranial bleeding seen after randomisation in patients who undergo neurosurgical haemorrhage evacuation.
(e) Composite poor outcome: progressive bleeding (“a” above), new bleeding (“b” above), cerebral infarction (“c” above), death or the need for neurosurgery within 28 days of injury.

All outcomes for patients treated after three hours of injury will be presented separately.

### Trial status

The first patient was enrolled in the CRASH-3 trial on 20 July 2012. Data collection for the CRASH-3 IBMS started in February 2016. The CRASH-3 trial completed recruitment on 31 January 2019. Routinely collected brain imaging data from patients included in the CRASH-3 IBMS were examined for the purpose of the IBMS and recorded in a web database before this date.

The first and second versions of this SAP were submitted for publication (and publicly available) well before the trials unblinded on 31 May 2019. The reviewer report from peer reviewer 4 was submitted on 6 June 2019. This third version of the SAP is in response to small requests for clarification from peer reviewer 4.

## Statistical analysis plan

### Trial profile

We will show the flow of trial patients in the Consolidated Standards of Reporting Trials (CONSORT) diagram. This will include the total number of patients randomised into the IBMS divided by treatment arm. Each treatment arm will detail the number of patients who received the loading and maintenance doses, the number of patients for whom clinical baseline and outcome data was collected, and the number of patients who were scanned before randomisation and/or after randomisation. We will report the number of patients included in the primary and secondary analyses, the reasons for any post-randomisation exclusions and the number lost to follow-up. If after a patient is randomised into the trial, it is found that they did not meet the eligibility criteria or did not receive their allocated treatment, they are considered to have deviated from the trial protocol. Data from patients who have deviated from the protocol will be included in the intention to treat analysis. If a patient or their representative withdraws consent for data collection, we will use only data up to the point of withdrawal in the analysis.

### Baseline characteristics

We will report baseline characteristics, including, age, sex, GCS, systolic blood pressure, mean number of hours from injury to pre-randomisation scan, mean (and median) haemorrhage volume, different types of haemorrhage (intra-parenchymal, intra-ventricular, subdural, epidural, subarachnoid and petechial), cerebral infarction, oedematous lesions, mass effect findings, and the Marshall classification ^18^. To check that randomisation produced similar groups, we will describe the baseline characteristics of each treatment group with frequencies and percentages.

### Primary analysis

Linear mixed model ^26^ will be used to compare the mean change in intra-parenchymal haemorrhage volume from pre- to post-randomisation between treatment groups. The basic model includes pre- and post-randomisation volumes as correlated outcomes with mean post-randomisation volumes allowed to differ by treatment group but mean pre-randomisation volumes constrained to be the same, and with variances of pre- and post-randomisation volumes allowed to differ. In the absence of missing data, this linear mixed model gives identical estimates of the treatment effect, and near identical standard errors to the more standard ANCOVA analysis. The advantage of the linear mixed model approach is that patients with missing pre- or post-randomisation scans can be included in the analysis, potentially reducing bias and increasing efficiency ^26^.

A linear regression analysis of the blinded data indicated that time from injury to CT scan, GCS, age and systolic blood pressure are significantly predictive of the pre- and post-randomisation bleeding volumes (p<0.05). These covariates and the stratification factor (hospital site) ^27^ will be included in the analysis to improve the precision of the effect estimate. The main effect of site (which is treated as fixed) is accounted for in the model. Because we have relatively few centres (n=14) compared to the number of patients (n= ≈1750), we expect any loss in efficiency from this method (compared to the random centre effects method) to be minimal. The linear mixed model described above will include an interaction between each covariate and whether bleeding volume was measured before and/or after randomisation. This gives treatment effect estimates that are identical to those from the standard ANCOVA model in the absence of missing data. We expect the covariates to affect bleeding volumes in different ways (e.g. older people are likely to have larger bleeds at baseline, more severely unconscious people (low GCS) are likely to have larger bleeds at baseline). In line with the CONSORT guidelines ^28^, we will also report the results from the linear mixed model without covariate adjustment to facilitate synthesis and comparability with other trials that may not include the same covariates.

The blinded data indicates that pre-randomisation and post-randomisation bleeding volumes are positively skewed. Because bleeding volumes are skewed, this data will be log transformed before entered into the linear mixed model. The anti-log of the treatment effect estimate and its corresponding 95% CIs will be presented to aid interpretation. The treatment effect estimates will provide an estimate of the relative increase or decrease in haemorrhage volume with tranexamic acid.

In the original protocol, we planned to analyse the primary outcome using ANCOVA. Since publishing the protocol, we learnt that less than 50% of patients were scanned both pre- and post-randomisation. Because the pre-randomisation mean bleeding volume of the observed data may be different from the true pre-randomisation mean bleeding volume, the estimates from the ANCOVA model may be biased. Compared to ANCOVA, linear mixed models are more powerful and typically less biased when there are missing data ^26^.

### Sensitivity analysis

*Exclude patients who underwent neurosurgical haemorrhage evacuation after randomisation:* The blinded data shows that after randomisation 14% of patients had neurosurgery before undergoing the first rated post-randomisation scan. In these cases, it is difficult to use the post-randomisation and post-neurosurgery scan to estimate the treatment effect because any change seen in intracranial haemorrhage expansion or infarction could be due to the effect of tranexamic acid or neurosurgery. The inclusion of these patients in the primary analysis may dilute any treatment effect towards the null. Therefore, we will conduct a sensitivity analysis excluding patients who underwent neurosurgery before a post-randomisation scan was done.

### Secondary analyses

*Composite poor outcome, progressive haemorrhage, new haemorrhage, haemorrhagic oedematous lesions and mass effect*: We will express the effect of tranexamic acid on the occurrence of dichotomous endpoints between trial arms, including the frequency of the composite “poor” outcome, progressive haemorrhage, new haemorrhage, haemorrhagic oedematous lesions, and mass effect outcomes (sulcal effacement, ventricular effacement, midline shift), using relative risks and 95% confidence intervals estimated using generalised linear models. We will express the effect of tranexamic acid on the degree of midline shift (measured in millimetres) using a basic linear mixed model, with pre-randomisation midline shift included as an outcome (as described above). We will extend this model to include covariates and their interaction with midline shift: time from injury to scan, GCS, age and systolic blood pressure.

*Cerebral infarction:* We will express the effect of tranexamic acid on cerebral infarcts measured at up to 28 days post-randomisation and not known to be present pre-randomisation using hazard ratios and 95% confidence intervals. We will conduct a survival analysis using the interval between the time of randomisation and the time of the scan on which the infarct was detected. We will plot the survival curves in the two treatment groups using a Kaplan-Meier plot. The time to the scan on which the infarct was detected will be compared between treatment groups using a log-rank test. We will conduct a Cox regression analysis to quantify any difference between treatment groups in the hazard of detecting an infarct up to 28 days post-randomisation. We will conduct a sensitivity analysis excluding the patients who underwent neurosurgery.

*Neurosurgical haemorrhage evacuation after randomisation*: If tranexamic acid received soon after injury reduces intracranial haemorrhage, a patient who received tranexamic acid may be less likely to undergo neurosurgery to evacuate haemorrhage compared with a patient who received placebo. However, in an emergency trauma setting, the decision for neurosurgery occurs at the same time or very soon after the time of randomisation. Therefore, tranexamic acid received soon after injury may not affect the propensity for neurosurgery. But it could affect intracranial bleeding during neurosurgery.

We hypothesise that patients who receive tranexamic acid may have less blood on a post-randomisation and post-neurosurgery scan compared with patients who receive placebo. We will express the effect of tranexamic acid on the total volume of intracranial haemorrhage measured on a post-randomisation and post-neurosurgery scan using a linear mixed model as above. If the patient has been scanned pre-randomisation (and pre-neurosurgery), we will include the pre-randomisation bleeding volume as a variable in the linear mixed model as above. To improve the precision of the effect estimate, we will extend this model to include each covariate and its interaction with bleeding volume: time from injury to scans, time from neurosurgery to scan, GCS, age and systolic blood pressure.

We will conduct a survival analysis using the time from randomisation to neurosurgery. The time to neurosurgery will be compared between treatment arms using a log-rank test. Because the log-rank test will only indicate whether there is a significant difference between treatment arms in the time to neurosurgery, we will also conduct a Cox regression analysis to quantify any difference in the hazard of neurosurgery between arms.

*Subarachnoid haemorrhage*: We will express the effect of tranexamic acid on the size (small-medium, large) and spread (focal-multiple, diffuse) of subarachnoid haemorrhage between trial arms, using relative risks and 95% confidence intervals estimated using generalised linear models.

### Subgroup analyses

*Time from injury to randomisation:* Most intracranial bleeding occurs within hours of injury ^5, 25, 29, 30^. Subgroup analyses will examine whether the effect of tranexamic acid on intracranial haemorrhage is modified by the time from injury to randomisation (≤1 hour, >1 to 3 hours, >3 to 8 hours). If there is minimal haemorrhage expansion after 3 hours ^25^, we expect tranexamic acid will have a lesser effect in reducing haemorrhage expansion in this group compared to the groups treated within 3 hours. We will conduct a linear regression analysis with an interaction between treatment (tranexamic acid, placebo) and time to randomisation (≤1 hour, 1–3 hours, >3–8 hours) to examine whether the effect of tranexamic acid on intracranial haemorrhage volume varies according to the time from injury to randomisation.

There may be an increase in the frequency of cerebral infarction with tranexamic acid in those treated after 3 hours of injury compared to those treated within 3 hours of injury ^15^. We will use relative risks and 95% confidence intervals estimated using generalised linear models to examine whether the effect of tranexamic acid on cerebral infarction varies within subgroups of time from injury to randomisation (≤3 hours, >3 hours). However, given the lower prevalence of cerebral infarction compared to intracranial bleeding, it will be difficult to reliably examine the effect of tranexamic acid on cerebral infarction within time strata. We will examine whether tranexamic acid increases the risk of adverse events in an individual patient data meta-analysis of 15,000 patients with TBI or spontaneous intracerebral haemorrhage (published separately) ^31^.

*Types of haemorrhage*: We will conduct the linear mixed model analysis specified in the primary analysis section separately for subdural, epidural and intra-ventricular bleeds.

### Missing data from scans not done before or after randomisation

Not all trial patients will be scanned before and after randomisation. We will report the number of patients without scans ^28^ and baseline data for patients included in the analysis to help identify any selective missingness of outcomes by treatment arm ^32^. We will examine whether missing scans are missing equally between treatment arms and appear to be missing completely at random (MCAR). In this case, although missing data reduces the precision of the analysis, it does not bias the treatment effect ^33^.

However, if haemorrhage expansion is associated with the reason the data are missing (patients with haemorrhage expansion may die before the second scan, patients without haemorrhage may not need to be re-scanned), imbalance in missing data by treatment arm can cause bias. We will examine whether the occurrence of missing scans is influenced by fully observed baseline variables (e.g. GCS), using relative risks and 95% confidence intervals estimated using generalised linear models. If they are, and within defined groups data are missing completely at random, the data could be missing at random (MAR) ^33^. For example, if missingness depends on GCS, but within mild, moderate and severe GCS groups missingness is unrelated to haemorrhage or infarction, the data are MAR. In this case, a regression analysis which takes GCS group into account should give unbiased estimates of the treatment effect ^34^.

However, we suspect that within GCS groups, missingness could be related to haemorrhage volume (i.e. low GCS patients are expected to have a greater haemorrhage volume than high GCS patients). In this case, the data would be missing not at random (MNAR) (i.e. even when accounting for the fully observed data, the reason for missing observations still depends on the unseen values) ^33^.

Because injury severity can partly explain missingness and there are unknown reasons for some missingness, it is difficult to confirm whether our missing data will be MAR or MNAR. For the purpose of the primary analysis, we will assume missing data are MAR. To examine how robust the primary analysis is to the chosen method of handling missing data, we will conduct sensitivity analyses assuming missing data are MCAR or MNAR. Under the MCAR assumption, we will compare haemorrhage volumes between treatment groups without accounting for missingness. Under the MNAR assumption, we will compare haemorrhage volumes between treatment groups and explore the possibility that missingness of the outcome data is related to prognostic characteristics as well as to the trial treatment. If tranexamic acid reduces intracranial haemorrhage expansion and the risk of death, patients who receive tranexamic acid may be more likely to be scanned post-randomisation compared to those who receive placebo. On the other hand, if tranexamic acid reduces or prevents intracranial haemorrhage expansion, post-randomisation scanning may not be clinically indicated in these patients. We will conduct sensitivity analyses excluding patients with a low pre-randomisation GCS who may have large haemorrhage expansion and therefore not survive to have a post-randomisation scan. We will conduct sensitivity analyses excluding patients with a high pre-randomisation GCS who may have smaller haemorrhage expansion and therefore not require a post-randomisation scan.

### Between-centre effects

Randomisation into the CRASH-3 trial is stratified according to participating centres. We do not expect between-centre differences in unfavourable outcome to affect the chance of demonstrating a treatment effect in TBI ^35^. Nonetheless, the main effect of site will be included in the analyses. 

## Conclusion

This statistical analysis plan updates our previously published protocol ^18^. The main changes are: an increased sample size from 1,000 to a maximum of 2,000 patients, a comparison of intra-parenchymal bleeding expansion between treatment groups for the primary outcome, the use of covariate adjusted linear mixed models for the primary analysis and relevant secondary analyses, and restriction of the analysis of the primary and secondary outcomes (new and progressive bleeding) to patients treated within three hours of injury. We present our plan for the statistical analyses in advance of the database lock and un-blinding to guard against data dependent analyses. The CRASH-3 IBMS should provide reliable evidence on the effect of tranexamic acid on intracranial bleeding and infarction in TBI.

## Data availability

No data are associated with this article.
