# Supplementary material for: A nested randomised trial of the effect of tranexamic acid on intracranial haemorrhage and infarction in traumatic brain injury (CRASH-3 trial intracranial bleeding mechanistic study): Statistical analysis plan
Source: Wellcome Open Res. 2019 Nov 27;3:99. Originally published 2018 Aug 13. [Version 3] doi: 10.12688/wellcomeopenres.14731.3 (PMC6530602; doi:10.12688/wellcomeopenres.14731.3)
Supplement: Peer review history

# Peer review: Reviewer response for version 3

**DOI:** 10.21956/wellcomeopenres.17099.r37173

**Published:** 2019-12-04

**Authors:** Christian Gluud

**Affiliations:** 1 The Copenhagen Trial Unit, Centre for Clinical Intervention Research, Copenhagen, Denmark

I have read the responses to my comments and are satisfied with them. I have also read the article again and finds it improved and easier to understand. I therefore approve the article as it is.

 During my read, I noticed the following minor things that you may decide on yourselves:

- LSHTM is not explained in the box under Grant information.
- In the box named revised, there ought to be a space between comments from reviewer 3 and from reviewer 4.
- Page 4, first column: LSHTM is not explained – but it is on p. 5.
- Page 4, second c: Full stop after 95% should come after the following parenthesis.
- Page 4, second c: double-blinded should become blinded.
- Page 5, second c: compared to placebo ought to become compared with placebo.
- Page 7, second c: 1-3 hours ought to become >1-3 hours.
  Page 8, first c: compared to those who receive placebo ought to become compared with those who receive placebo.

I confirm that I have read this submission and believe that I have an appropriate level of expertise to confirm that it is of an acceptable scientific standard.

# Peer review: Reviewer response for version 2

**DOI:** 10.21956/wellcomeopenres.16363.r35534

**Published:** 2019-06-06

**Authors:** Christian Gluud

**Affiliations:** 1 The Copenhagen Trial Unit, Centre for Clinical Intervention Research, Copenhagen, Denmark

Mahmood and colleagues have developed a statistical analysis plan for their nested CRASH-3 randomised clinical trial assessing tranexamic acid versus placebo for people with intracranial haemorrhage in traumatic brain injury. The primary outcome is the volume of intra-parenchymal bleeding in participants randomised within three hours of injury. The patients included will be up to 2000 participants out of the total of almost 13,000 participant randomised in the CRASH-3 trial which ended early 2019. The trial is of utmost importance. So is this nested sub-study, dealing with the population that were the only population randomised in the trial from 10 ^th^ of November 2015. The statistical analysis plan is well written and clear. I only read the version 2, which has undergone amendments since first publication in August 2018. However, I have some points where it is difficult for me to fully understand the plan.

 My suggestions for further explanations or clarity are: 

1. The sample size estimation may still have some problems. When one has a volume, it is like a continuous outcome, giving the best power. As the data are skewed, I understand the logarithmic transformation. But why then dichotomise the transformed data? Is it easier for the reader to understand? Or is it in order to calculate a number needed to treat? Then I maybe understand a little. Usually, I would recommend to use the original volume in mL for the calculation of the sample size based on the assumed minimal relevant difference as well as a plausible standard deviation. Moreover, if you really want to dichotomise it, then you need to give a proportion in the control group having a bleed larger than e.g. three mL, and then take your relative risk reduction or increase based on that. As I see it this control proportion is missing.
2. On p 5, the authors say they will adjust their analysis for prognostic factors. I see no mention of site here. Moreover, the selection of the prognostic factors going into the analysis could become clearer?
3. On p.6, participants that are operated become a sensitivity analysis, whereas earlier them were presented as the primary analysis?
4. On p.7, pre-randomisation bleeding volume is called ’an outcome’. Should that not become a ‘variable’?
5. On p. 8 MAR, MNAR, and MCAR are used extensively. But I am not sure how to interpret the likely multiple differing outcomes. Maybe, the potential impact of missingness could be examined by just applying ‘best-worst’ and ‘worst-best’ scenario analyses?
6. In the title and in the Abstract, I lack information on the fact that you examine the effect of tranexamic acid versus placebo. This is a central advantage of this trial that can only be mention too seldomly.
7. In the Abstract, the primary outcome is said to be the volume, which seems to contrast with the sample size calculation (see point 1).
8. On p. 3, it says: "in a meta-analysis, there was a statistically significant reduction in intracranial haemmorhage growth". Considering that the reader do not know the bias risks of the trials and that the confidence interval is the naïve 95%, maybe it could be formulated a bit weaker? E.g., "in a meta-analysis, there seemed to be a reduction in"?
9. The present status of the trial needs to become clearer. As I understand it, all randomisation has stopped earlier this year?  
10. On p 7, first column, lower third. Here ‘compared to’ should become ‘compared with’?
11. I understand that this SAP has been submitted during 2018, well before randomisation was stopped and data examined. This also needs to be clearly discussed in the light that the trial has now sized randomisation and the data likely been analysed?
12. The alpha level chosen for this analysis of the primary outcome is 0.05. As this is an extra analysis, one could have chosen a more stringent level to keep the type 1 family wise error under 0.05. This needs to be discussed.
13. The remaining statistical analyses including subgroup and sensitivity analyses all see also to be conducted at the alpha level of 0.05. This is likely ok but should one not then stress that all these analyses will be viewed as exploratory analyses due to the high risks of type I errors?

I confirm that I have read this submission and believe that I have an appropriate level of expertise to confirm that it is of an acceptable scientific standard, however I have significant reservations, as outlined above.

# Response

**Published:** 2019-11-21

**Authors:** Abda Mahmood

**Affiliations:** London School of Hygiene & Tropical Medicine, UK

**Author response:**

Thank you for your detailed review of the analysis plan for the CRASH-3 IBMS. We have responded to the below queries and hope this makes the analysis plan clearer. Please note that we received this report after the trial unblinded, and are not able to make substantial changes to the analysis plan at this stage.

**Reviewer comment:**

My suggestions for further explanations or clarity are: 

- The sample size estimation may still have some problems. When one has a volume, it is like a continuous outcome, giving the best power. As the data are skewed, I understand the logarithmic transformation. But why then dichotomise the transformed data? Is it easier for the reader to understand? Or is it in order to calculate a number needed to treat? Then I maybe understand a little. Usually, I would recommend to use the original volume in mL for the calculation of the sample size based on the assumed minimal relevant difference as well as a plausible standard deviation. Moreover, if you really want to dichotomise it, then you need to give a proportion in the control group having a bleed larger than e.g. three mL, and then take your relative risk reduction or increase based on that. As I see it this control proportion is missing.

**Author response:**

The relevant section of the SAP is: “The blinded data indicates that pre- and post-randomisation bleeding volumes are positively skewed. We will log transform these values and report the primary outcome as a proportion.”

There has been some confusion in interpretation, probably because of a lack of clarity on our part. We do not plan to dichotomise the log transformed bleeding volume. Bleeding volume is continuous and measured in millilitres (ml). Because bleeding volumes are skewed, this data will be log transformed before entered into the linear mixed model. The anti-log of the treatment effect estimate and its corresponding 95% confidence intervals (CIs) will be presented to aid interpretation. The treatment effect estimates will provide an estimate of the relative increase or decrease in haemorrhage volume with tranexamic acid. The text in the manuscript has been amended for clarity.

This does not affect the sample size calculation.

**Reviewer comment:**

- On p 5, the authors say they will adjust their analysis for prognostic factors. I see no mention of site here. Moreover, the selection of the prognostic factors going into the analysis could become clearer?

**Author response:**

We stated in the primary analysis section that Site will be included in the model.

We clarified in response to reviewer 3’s query that the main effect of site is accounted for in the primary analysis.

The selection of prognostic factors is based on the established association between time from injury to scan, GCS, age and systolic blood pressure on bleeding volume. The blinded data support this association and so these covariates will be included in the model. This is noted in the primary analysis section.

**Reviewer comment:**

- On p.6, participants that are operated become a sensitivity analysis, whereas earlier them were presented as the primary analysis?

**Author response:**

The primary analysis includes all patients and its sensitivity analysis excludes patients who underwent neurosurgical haemorrhage evacuation. A secondary analysis looks at the effect of tranexamic on bleeding in patients who underwent neurosurgical haemorrhage evacuation.

**Reviewer comment:**

- On p.7, pre-randomisation bleeding volume is called ’an outcome’. Should that not become a ‘variable’?

**Author response:**

In the mixed model literature, pre-randomisation data is often referred to as an “outcome”. We understand that this can be confusing as it is not measured after randomisation, and so we will change this to “variable” as suggested.

**Reviewer comment:**

- On p. 8 MAR, MNAR, and MCAR are used extensively. But I am not sure how to interpret the likely multiple differing outcomes. Maybe, the potential impact of missingness could be examined by just applying ‘best-worst’ and ‘worst-best’ scenario analyses?

**Author response:**

Thank you for your suggestion. We tried to methodically explain why we don’t think missing data will be from a random subset of trial patients (i.e. it will not be missing completely at random), it may not be fully explained by baseline prognostic characteristics (i.e. it may not be missing at random), but it may be related to prognostic characteristics as well as to the trial treatment (i.e. it may be missing not at random). Your suggestion for applying best-worst and worst-best sensitivity analyses is helpful, and we will do this to help assess the impact of missingness on effect estimates. But a large proportion of patients were not scanned post-randomisation in the CRASH-3 IBMS, and so best-worst and worst-best scenarios may merely indicate the best case scenario (benefit with trial treatment) and worst case scenario (harm with trial treatment) by definition of how the missing values are imputed. For these outcomes, the results of the complete case analyses may be more useful, in the context of a clear discussion of the resulting interpretative limitations of missing post-randomisation scans (Jakobsen *et al*., 2017).

Jakobsen JC, Gluud C, Wetterslev J, Winkel P. When and how should multiple imputation be used for handling missing data in randomised clinical trials - a practical guide with flowcharts. Bmc Medical Research Methodology. 2017 Dec 6;17

**Reviewer comment:**

- In the title and in the Abstract, I lack information on the fact that you examine the effect of tranexamic acid versus placebo. This is a central advantage of this trial that can only be mention too seldomly.

**Author response:**

We have amended the abstract accordingly.

**Reviewer comment:**

- In the Abstract, the primary outcome is said to be the volume, which seems to contrast with the sample size calculation (see point 1).

**Author response:**

Please see our response to point 1.

**Reviewer comment:**

- On p. 3, it says: "in a meta-analysis, there was a statistically significant reduction in intracranial haemmorhage growth". Considering that the reader do not know the bias risks of the trials and that the confidence interval is the naïve 95%, maybe it could be formulated a bit weaker? E.g., "in a meta-analysis, there seemed to be a reduction in"?

**Author response:**

We have amended this accordingly.

**Reviewer comment:**

- The present status of the trial needs to become clearer. As I understand it, all randomisation has stopped earlier this year?  

**Author response:**

Yes, the trial finished recruitment on 31 January 2019 and unblinded on 31 May 2019. The “trial status” section has been updated for clarity.

**Reviewer comment:**

- On p 7, first column, lower third. Here ‘compared to’ should become ‘compared with’?

**Author response:**

This has been amended accordingly.

**Reviewer comment:**

- I understand that this SAP has been submitted during 2018, well before randomisation was stopped and data examined. This also needs to be clearly discussed in the light that the trial has now sized randomisation and the data likely been analysed?

**Author response:**

The analysis plan was submitted and published before the trial unblinded. We received this referee report after the trial unblinded. We have not made any substantial changes to the analysis plan as per this review. We only respond to acknowledge and address the queries raised, and update the relevant sections of the SAP for clarity as requested. The “trial status” section has been updated to clarify.

**Reviewer comment:**

- The alpha level chosen for this analysis of the primary outcome is 0.05. As this is an extra analysis, one could have chosen a more stringent level to keep the type 1 family wise error under 0.05. This needs to be discussed.

**Author response:**

Although this trial is nested within the CRASH-3 trial, it has a different purpose. The CRASH-3 trial examines the effect of tranexamic acid versus placebo on death and disability in TBI patients, whereas the mechanistic study examines intracranial bleeding and other neuro-radiological characteristics in these patients.

We will consider the implications of type 1 error when interpreting the results and include this in the discussion of any results publication.

**Reviewer comment:**

- The remaining statistical analyses including subgroup and sensitivity analyses all see also to be conducted at the alpha level of 0.05. This is likely ok but should one not then stress that all these analyses will be viewed as exploratory analyses due to the high risks of type I errors?

**Author response:**

We acknowledge that we are examining the effect of the trial treatment on a number of endpoints and so there is a high risk of type 1 error. We will consider the implications of type 1 error when interpreting the results and stress that these analyses are exploratory in the final results publication.

Many thanks for your thorough review.

# Peer review: Reviewer response for version 2

**DOI:** 10.21956/wellcomeopenres.16363.r35529

**Published:** 2019-05-21

**Authors:** Stephen Senn

**Affiliations:** 1 Independent Consultant Statistician, Strassen, Luxembourg

**Introduction**

 This is a careful and detailed description of an important clinical trial. I claim no knowledge of the medical specialty and, as befits a statistician, limit myself to a discussion of statistical aspects only.

**Power calculation**

 The acid test is “can the calculation be repeated?” Unfortunately, the answer is “no”. What is missing is the standard deviation of the reduction. I calculate that if the SD were 87 for an effect of 15 then about 1750 patients would be needed to give 95% power and that a reduction of sample size to 1300 would have about 87% power. However, a standard deviation that is 87% of the mean control value size is very large and implies a lack of Normality, which in turn suggest that a log-transformation might be needed. In fact, later we encounter the statement: " *The blinded data indicates that pre- and post-randomisation bleeding volumes are positively skewed. We will log transform these values and report the primary outcome as a proportion*.” Whatever the explanation, the necessary detail is lacking.

**Statistical analysis**

 This is a multi-centre trial. Reference is made to a treatment by centre interaction being investigated but the reference to modelling the main effect of centre is imprecise; it is stated “ *stratification factor (treatment site) 27 will be included in the analysis to improve the precision of the effect estimate*”, which uses terminology that is inconsistent with that used for interaction. The plan does not say how the main effect of centres will be allowed for. There are two standard ways to include the main effect of centre in the model. One is to treat the centre effect as fixed and the other as random ^1^. If there are many small centre and if there is some imbalance, the former may be inefficient. The latter requires care when covariates are involved because regression terms should, in theory, be allowed for at two levels: both between and within centres. An analogous problem occurs in cross-over trials. A useful reference is that of Kenward and Roger ^2^.

 Also, it is not clear to me what this statement means: “ *This will be done by extending the basic linear mixed model described above to include each covariate and its interaction with bleeding volume (pre- versus post-randomisation).*”

I confirm that I have read this submission and believe that I have an appropriate level of expertise to confirm that it is of an acceptable scientific standard.

## References

[1] : A note regarding ‘random effects’. *Statistics in Medicine*.2014;33(16) : 10.1002/sim.5965 2876-2877 10.1002/sim.5965 24898710

[2] : The use of baseline covariates in crossover studies. *Biostatistics*.2010;11(1) : 10.1093/biostatistics/kxp046 1-17 10.1093/biostatistics/kxp046 19915170

# Response

**Published:** 2019-11-21

**Authors:** Abda Mahmood

**Affiliations:** London School of Hygiene & Tropical Medicine, UK

**Reviewer: Introduction**

This is a careful and detailed description of an important clinical trial. I claim no knowledge of the medical specialty and, as befits a statistician, limit myself to a discussion of statistical aspects only.

**Reviewer: Power calculation**

The acid test is “can the calculation be repeated?” Unfortunately, the answer is “no”. What is missing is the standard deviation of the reduction. I calculate that if the SD were 87 for an effect of 15 then about 1750 patients would be needed to give 95% power and that a reduction of sample size to 1300 would have about 87% power. However, a standard deviation that is 87% of the mean control value size is very large and implies a lack of Normality, which in turn suggest that a log-transformation might be needed. In fact, later we encounter the statement: " *The blinded data indicates that pre- and post-randomisation bleeding volumes are positively skewed. We will log transform these values and report the primary outcome as a proportion*.” Whatever the explanation, the necessary detail is lacking.

**Author response**

Thank you for your comment. The sample size calculation has been clarified below, and we hope this now passes the acid test.

We originally planned for the IBMS to be conducted in 1,000 CRASH-3 trial patients. This sample size was based on the reduction in bleeding volume seen with tranexamic acid in the CRASH-2 Intracranial Bleeding Sub-study (Perel *et al*., 2012). We expected a 15% reduction in intracranial bleeding with tranexamic acid (24ml tranexamic acid, 28ml placebo), a correlation of 0.6 between pre- and post-randomisation bleeding volumes, and a standard deviation of 28ml. This gave an unadjusted sample size estimate of 1542, which was reduced to 987 with adjustment (1542*(1-(0.6 ^2^)) (Borm *et al*., 2007).

Due to the large amount of missing data (only around half of patients were scanned both pre-randomisation and post-randomisation), we increased the sample size for the CRASH-3 IBMS to include a maximum of 2000 patients. This was the approximate maximum number of patients the scan assessor could feasibly collect data from before the CRASH-3 trial completed recruitment. It was not expected that the scan assessor would be able to collect data from 2,000 patients (due to many international sites not using electronic imaging, and the limited time and resources for this study). This upper bound was chosen to prevent delays in data collection as a result of protocol amendments that would be needed should the sample size be increased again.

More realistically, we expected around 1,700 patients could be included in the CRASH-3 IBMS. Using the same expected treatment effect, standard deviation, correlation and baseline adjustment values as the original sample size calculation, this increased power to 95%. If only considering those treated within 3 hours of injury (n=1300), there is 90% power to detect the expected treatment effect. However, this calculation is not adjusted for missing data. If we assume that 47% of patients will be dropped from the analyses, this leaves a study with 901 patients scanned both pre-randomisation and post-randomisation. Using the same standard deviation (adjusted for baseline), correlation and baseline adjustment values as the original sample size calculation, a study with 901 patients would have 76% power to detect the expected treatment effect. In case it is helpful, the relevant Stata code is below:

***Original calculation***

power twomeans 24 28, sd(28) // sample size estimate (n=1542)

di 1542*(1-0.6^2) // adjusted sample size estimate (n=987)

***Variance deflation factor adjustment***

di sqrt(28^2*(1-0.6^2))  // SD=22.4

***Estimated power of expected sample size***

power twomeans 24 28, sd(22.4) n(901) // 76% power

The sample size section of the SAP has been amended.

**Reviewer: Statistical analysis**

This is a multi-centre trial. Reference is made to a treatment by centre interaction being investigated but the reference to modelling the main effect of centre is imprecise; it is stated “ *stratification factor (treatment site) will be included in the analysis to improve the precision of the effect estimate*”, which uses terminology that is inconsistent with that used for interaction. The plan does not say how the main effect of centres will be allowed for. There are two standard ways to include the main effect of centre in the model. One is to treat the centre effect as fixed and the other as random ^1^. If there are many small centre and if there is some imbalance, the former may be inefficient. The latter requires care when covariates are involved because regression terms should, in theory, be allowed for at two levels: both between and within centres. An analogous problem occurs in cross-over trials. A useful reference is that of Kenward and Roger ^2^.

**Author response:**

Thank you for your detailed comment. The specified linear mixed model analyses, which compare bleeding volume between treatment groups, include an interaction between centre and whether bleeding volume was measured before and/or after randomisation. Therefore, the main effect of centre (which is treated as fixed) is accounted for in the model. Because we have relatively few centres (n=14) compared to the number of patients (n= ≈1750), we expect any loss in efficiency from this method (compared to the random centre effects method) to be minimal.

Although we noted in the SAP that we would include an interaction between centre and treatment in the model, we do not expect between centre differences in unfavourable outcome to affect the chance of demonstrating a treatment effect (Lingsma *et al*., 2011). There is also low power for a between-centre interaction. Therefore, we will not include the interaction between centre and treatment in the model. The relevant sections of the SAP have been amended.

**Reviewer comment:**

Also, it is not clear to me what this statement means: “ *This will be done by extending the basic linear mixed model described above to include each covariate and its interaction with bleeding volume (pre- versus post-randomisation).*”

**Author response:**

In linear mixed model analyses, which compare bleeding volume between treatment groups, we have pre-specified an interaction between each covariate and whether bleeding volume was measured before and/or after randomisation. The “basic linear model” does not include covariates. The model we will use for the primary analysis and relevant secondary analyses include covariates. The relevant section of the SAP has been amended. We hope this is clearer.  

**References**

Perel P, Al-Shahi Salman R, Kawahara T, Morris Z, Prieto-Merino D, Roberts I, et al. CRASH-2 (Clinical Randomisation of an Antifibrinolytic in Significant Haemorrhage) intracranial bleeding study: the effect of tranexamic acid in traumatic brain injury--a nested randomised, placebo-controlled trial. Health Technol Assess. 2012;16(13):iii-xii, 1-54

Borm GF, Fransen J, Lemmens WA. A simple sample size formula for analysis of covariance in randomized clinical trials. J Clin Epidemiol. 2007 Dec;60(12):1234-8

Lingsma HF, Roozenbeek B, Perel P, *et al.*: Between-centre differences and treatment effects in randomized controlled trials: a case study in traumatic brain injury. *Trials.* 2011; **12**: 201.

# Peer review: Reviewer response for version 1

**DOI:** 10.21956/wellcomeopenres.16049.r33944

**Published:** 2018-10-18

**Authors:** John Hodsoll

**Affiliations:** 1 Biostatistics Department, National Institute for Health Research (NIHR) Biomedical Research Centre for Mental Health, Institute of Psychiatry, Psychology & Neuroscience (IoPPN), King's College London, London, UK

The paper is clearly written and overall I would consider the analysis plan for this study appropriate. Specific suggestions and points that could be clarified are as follows:

1. What was the reason for 80% power rather than 90% (as is typical in a trial)? If this is justified in the protocol that is fine, but could be referred to in the present paper.
2. In the section on interim analyses it is stated no interim analysis is planned. However, in the primary analysis section (p5) a 1000 blinded subset of data was used to identify predictors of brain volume. I would have expected these to be defined a priori. Is there a justification / precedent for identifying candidate predictors as the authors have?
3. In terms of missing data, if there are many missing pre-randomisation scans it would be possible to include baseline as an outcome in a repeated measures linear mixed model, cf. Dinh & Yang (2010). The advantage of this approach is that linear mixed models can estimate the maximum likelihood function over missing (and non-missing) data and so subjects with either missing baseline or outcome scans could be included in the analysis.  Treatment effects are defined by an interaction between treatment arm and time.
4. Could the authors elaborate on the proposed sensitivity analysis relative to the MNAR assumption.
5. On p3 in the 2 ^nd^ paragraph of the introduction, the p-value for reference 11 is quoted as 1.17 which is greater than 1.

I confirm that I have read this submission and believe that I have an appropriate level of expertise to confirm that it is of an acceptable scientific standard, however I have significant reservations, as outlined above.

# Response

**Published:** 2018-12-18

**Authors:** Abda Mahmood

**Affiliations:** London School of Hygiene & Tropical Medicine, UK

Thank you for your valuable feedback on our paper. We have responded to each of your points below and all relevant changes have been incorporated into the revised version of the manuscript (version 1.1).

**What was the reason for 80% power rather than 90% (as is typical in a trial)? If this is justified in the protocol that is fine, but could be referred to in the present paper.**

 In the published protocol, we said that a trial with at least 1000 patients will have 80% power (at alpha = 0.05) to detect a 15% lower bleeding volume in the tranexamic acid group at follow-up (i.e., 24 mL tranexamic acid vs. 28 mL placebo). This was based on the treatment effect observed in a smaller randomised trial of tranexamic acid in traumatic brain injury (CRASH-2 Trial Intracranial Bleeding Study; Perel et al., 2012). Since publishing the protocol, we increased the sample size from 1,000 to around 1,750 patients, and thereby the power from 80% to 95%, which will be further improved by covariate adjustment.

 Because the primary analysis will be based on patients randomised within 3 hours of injury, of which at the time of writing the first version of the SAP we expected there to be 1000, we reported the power of the primary analysis to detect the expected treatment effect. However, the power of the total sample is greater than this. We have amended the SAP to clarify (page 8).

**In the section on interim analyses it is stated no interim analysis is planned. However, in the primary analysis section (p5) a 1000 blinded subset of data was used to identify predictors of brain volume. I would have expected these to be defined a priori. Is there a justification / precedent for identifying candidate predictors as the authors have?**

 We plan to adjust the primary analysis using appropriately selected prognostic covariates. Time from injury to scan, age, GCS and systolic blood pressure have been shown to predict intracranial haemorrhage volume (Narayan et al., 2008; Yadav et al., 2006). We used our blinded data from 1000 patients to examine whether this finding was replicated in our trial because adjusting for non-prognostic covariates can lead to a reduction in power (Kahan et al., 2014). We found that the selected fully observed covariates are predictive of intracranial haemorrhage volume so we pre-specified that we will adjust for these covariates to improve the precision of the effect estimate.

**In terms of missing data, if there are many missing pre-randomisation scans it would be possible to include baseline as an outcome in a repeated measures linear mixed model, cf. Dinh & Yang (2010). The advantage of this approach is that linear mixed models can estimate the maximum likelihood function over missing (and non-missing) data and so subjects with either missing baseline or outcome scans could be included in the analysis. Treatment effects are defined by an interaction between treatment arm and time.**

 Thank you for suggesting this alternative more powerful approach. We will use linear mixed models for the primary analysis and relevant other analyses, as specified in the updated version of the SAP.

**Could the authors elaborate on the proposed sensitivity analysis relative to the MNAR assumption.**

 We have updated the relevant section of the SAP accordingly (page 18).

**On p3 in the 2 ^nd^ paragraph of the introduction, the p-value for reference 11 is quoted as 1.17 which is greater than 1.**

 Thank you for picking up this typo. The p-value has been corrected to 0.17 (page 3).

**References**

 Narayan RK, Maas AI, Servadei F, Skolnick BE, Tillinger MN, Marshall LF, et al. Progression of traumatic intracerebral hemorrhage: a prospective observational study. *J Neurotrauma*. 2008;25(6):629-39.

 Kahan BC, Jairath V, Dore CJ, Morris TP. The risks and rewards of covariate adjustment in randomized trials: an assessment of 12 outcomes from 8 studies. Trials. 2014;15:139.

 Perel P, Al-Shahi Salman R, Kawahara T, Morris Z, Prieto-Merino D, Roberts I, et al. CRASH-2 (Clinical Randomisation of an Antifibrinolytic in Significant Haemorrhage) intracranial bleeding study: the effect of tranexamic acid in traumatic brain injury--a nested randomised, placebo-controlled trial. Health Technol Assess. 2012;16(13):iii-xii, 1-54.

 Yadav YR, Basoor A, Jain G, Nelson A. Expanding traumatic intracerebral contusion/hematoma. Neurol India. 2006;54:377-81.

# Peer review: Reviewer response for version 1

**DOI:** 10.21956/wellcomeopenres.16049.r33681

**Published:** 2018-09-17

**Authors:** Surakrant Yutthakasemsunt

**Affiliations:** 1 Department of Surgery, Khon Kaen Hospital, Khon Kaen, Thailand

This is an interesting study to verify relevant clinical contexts with reference to the pre-specified statistical analysis. We could not investigate the mechanism of underlying intracranial bleeding directly by therapeutic trial design of both CRASH3 and this study. However it could help for exploring and generating hypothesis about mechanisms of pharmacological action by different statistical plan in the study patients. In my opinion, the analytical plan of CRASH3 trial and related studies ^1^^-^^3^are comparable to the concept in meta analysis that exploring the clinical heterogeneity and statistical heterogeneity ^4^^-^^5^ among the studies of antifibrinolytic treatment for acute traumatic brain injury by the finding of reporting evidences. I look forward to seeing the result and encourage to continue such workings hereby. Finally, the concordant result among studies including explorative details in both treatment and control groups could have more evidences for traumatic intracranial bleeding.

I confirm that I have read this submission and believe that I have an appropriate level of expertise to confirm that it is of an acceptable scientific standard.

## References

[1] : Tranexamic acid for significant traumatic brain injury (The CRASH-3 trial): Statistical analysis plan for an international, randomised, double-blind, placebo-controlled trial. *Wellcome Open Res*.2018;3: 10.12688/wellcomeopenres.14700.1 86 10.12688/wellcomeopenres.14700.1 30175246PMC6081978

[2] : A nested mechanistic sub-study into the effect of tranexamic acid versus placebo on intracranial haemorrhage and cerebral ischaemia in isolated traumatic brain injury: study protocol for a randomised controlled trial (CRASH-3 Trial Intracranial Bleeding Mechanistic Sub-Study [CRASH-3 IBMS]). *Trials*.2017;18(1) : 10.1186/s13063-017-2073-6 10.1186/s13063-017-2073-6 28716153PMC5513059

[3] : The effectiveness and safety of antifibrinolytics in patients with acute intracranial haemorrhage: statistical analysis plan for an individual patient data meta-analysis. *Wellcome Open Res*.2017;2: 10.12688/wellcomeopenres.13262.2 120 10.12688/wellcomeopenres.13262.2 29417096PMC5785711

[4] : Why sources of heterogeneity in meta-analysis should be investigated. *BMJ*.1994;309(6965) :1351-5 786608510.1136/bmj.309.6965.1351PMC2541868

[5] : Explaining heterogeneity in meta-analysis: a comparison of methods. *Stat Med*.1999;18(20) :2693-708 1052186010.1002/(sici)1097-0258(19991030)18:20<2693::aid-sim235>3.0.co;2-v

# Response

**Published:** 2019-01-08

**Authors:** Abda Mahmood

**Affiliations:** London School of Hygiene & Tropical Medicine, UK

Thank you for reviewing our paper and for your thoughtful comments. We look forward to sharing the results in the near future!
